# Understanding the Sociocultural Dynamics of Loneliness in Southern Spanish Youth

**DOI:** 10.1007/s11013-024-09861-9

**Published:** 2024-06-12

**Authors:** Verónica C. Cala, Francisco Ortega

**Affiliations:** 1 Research Methods Department, University of Almeria, Crtra sacramento s/n 04120 (2.11 office, central building), Almería, Spain; 2 Medical Anthropology Research Center (MARC), Universitat Rovira i Virgili, Tarragona, Spain; 3 Catalan Institution for Research and Advanced Studies (ICREA), Barcelona, Spain

**Keywords:** Anthropology of loneliness, Loneliness, Sociocultural dynamics, Youth

## Abstract

Loneliness among young people has been increasing in recent years and is considered a major public health problem. This article delves into the sociocultural dynamics that favour the experiences of loneliness. For this purpose, 40 students between 19 and 24 years of age were interviewed using the photo elicitation interview (PEI) strategy. The results show a gradual normalization of the experience of loneliness and an effort to become accustomed to it. Virtual relationships and isolation linked to the COVID-19 pandemic are considered the two factors that have most enabled a climate prone to loneliness. Young people identify a few elements that feed social loneliness, such as an understanding of instrumental relationships, a scarcity of intimate relationships, a demand for hyperconnectivity, a fantasy of independence and a culture of positivity that hinders the establishment of quality social ties. Faced with hostile relational conditions, youth are sent into a cycle of loneliness. The greater the distrust of the environment is, the greater the defensive reactions and social distancing, and the greater the search for nearby spaces of refuge, security and shelter. Social withdrawal makes in-person relationships difficult and strengthens the need to isolate and become accustomed to loneliness.

## Introduction

Loneliness is a cross-cultural experience that has been described throughout history. For a few decades, it has been recognized as one of the most important health-related problems in Western societies (Cacioppo et al., 2014; Gerst-Emerson & Jayawardhana, 2015; Holt-Lunstad, 2017; Killeen, 1998). In 2018, the British government developed a strategy to address loneliness in the elderly population (Department for Digital, Culture, Media and Sport of British Government, 2018), which opened a public and media debate on the impact of the so-called “epidemic of loneliness” in western societies. In 2020, with the arrival of the COVID-19 pandemic, studies on loneliness rapidly increased, and concern spread globally until the phenomenon of loneliness became one of the main health problems according to the WHO (Jones, et al., 2021; WHO, 2021).

Although loneliness can be experienced at any age (Heinrich & Gullone, 2006), until the arrival of the pandemic, most studies focused on groups with special vulnerabilities, such as the elderly population and, to a lesser extent, migrants and single people. Research on young people was less considered until approximately 2015 (Fox, 2019). Interest in the collective has been growing as the incorporation of technologies has become more evident. Nevertheless, the meta-analysis by Surkalim et al. (2022) revealed that young people are still the age group for which the least statistical information is available. Prevalence studies show that loneliness is experienced by a considerable number of young people, with figures trending upwards. According to the AVIVA survey (2014), 53% of British people between 18 and 24 years old felt depressed due to loneliness. Qualter et al. (2015) reported that up to 80% of British young people have claimed to feel lonely at some point, and van Dulmen and Goossens (2013) reported that 22% of their sample population chronically experienced loneliness. More recently, a report by Baarck et al. (2021) indicated that after the pandemic, loneliness increased from 9% of European young people in 2016 to 35% in 2020, indicating that the affected population quadrupled.

Although loneliness is a fundamental human experience that can affect all people without an association with other pathologies (Ozawa-de Silva & Parsons, 2020), its impact on well-being and health cannot be ignored. There are numerous studies that associate it loneliness with, among other factors, an increase in psychiatric pathologies, decreased quality of life, increased risk behaviours (addictions and sedentary lifestyles) and suicide (Beutel et al., 2017; Chang et al., 2017; Macdonald et al., 2018).

### The Rise of the Anthropology of Loneliness

One of the difficulties of investigating loneliness lies in the complexity of providing a fixed definition of this experience. This is why a multitude of approaches have been offered from different disciplines, although psychology has dedicated more in-depth study to this reality, giving rise to various currents that range from psychodynamic and phenomenological angles predominant in the 1950s–60s (Fromm-Reichmann, 1957; Sullivan, 1953; Winnicott, 1958; Weiss, 1975) to more existential approaches in the 1970s, which recognize the need to address loneliness as a fundamental experience inherent to life (Mijuskovik, 1977; Moustakas, 1961). Since the 1980s, more cognitive (Jong Gierveld, 1998; Peplau & Perlman, 1982), neuroscientific and evolutionary approaches have been used (Cacioppo et al., 2014), and since the 2000s, multidimensional approaches are used (Stein & Tuval-Mashiach, 2015). Among the existing definitions, one of the most popular is that of Peplau and Pearlman (1982), who describe loneliness as an unpleasant experience caused by the perceived imbalance or deficiency between desired and real relationships. However, this cognitive and neuroevolutionary conceptions, hegemonic today, have received criticism for presenting loneliness as a merely individual, mental and subjective emotion separated from the body and the environment (Jenkins et al., 2020; Motta, 2021; Parsons, 2022). Similarly, it is discussed to what extent youth loneliness is an inherent episode of this period of life itself or is part of a social context that promotes it (Blakemore & Mills, 2014)

For this reason, at the height of the COVID-19 pandemic, some anthropologists, such as Ozawa-de Silva and Parsons (2020), defended the need to analyse loneliness not as a psychological or individual issue but as a social, cultural and relational phenomenon affecting the length and breadth of the planet. Given that expressions of loneliness occur in different ways and forms, loneliness needs to be culturally analysed. These researchers, influenced by Biehl et al. (2007), argue for the need to understand loneliness as a collective subjectivity modelled by historical-cultural circumstances. Previously, this holistic approach was also supported by Le Breton in his anthropological analysis of emotions:

Feelings or emotions are in no way purely physiological or psychological phenomena and are not left to chance or to the personal initiative of each actor. […] Feelings or emotions participate in a system of meanings and values of a social group, whose well-founded character confirms, as well as the principles that organize the social bond (Le Breton, 1999, pp. 11–12).

Recently, studies on paradigmatic cases, contexts and communities of loneliness, such as those carried out with hikikomori in Japan (Ismail, 2020; Ozawa da Silva, 2020; Saito, 2013), in post-natural catastrophe situations (Gagné, 2020), with Tuaregs in the Sahara Desert (Rasmussen, 2020), in migratory processes in cross-border contexts or with wild or abandoned children, shows different understandings and meanings of loneliness that reinforce the need to deepen the understanding of sociocultural frameworks and the specific meanings of this experience in each case.

### Internet Youth Cultures, Relational Transformations and Loneliness

Several studies have found a correlation between the increase in loneliness among Western youth and the emergence of youth digital cultures in neoliberal societies. The increase in youth loneliness, which is closely related to the incorporation of smartphones into young people’s lives and the increase in their use linked to the Covid-19 pandemic (d’Hombres et al., 2021; Kannan & Veazie, 2022), has engendered controversy regarding the role played by technologies in youth loneliness (Fox, 2019). Some authors argue that technologies are beneficial for coping with loneliness (Döring et al., 2022), while others claim that these are a source of loneliness. Since the 1990s, many authors have recognised that the internet’s role under neo-liberalism has impacted people’s modes of relationship, in addition to affecting relational intimacy (Illouz, 2007; Sibilia, 2008; Zafra, 2010), which refers to a person’s degree of affective connection, mutuality and reciprocity with other people (Amezaga et al., 2022; Turkle, 2015). Delafontaine et al. (2023) and Jansson et al. (2022) state that the lack of intimacy in relationships and the lack of meaning in life are central to the experience of loneliness.

Understanding socioeconomic and technological transformations, as well as how these affect modes of relationship and interaction, is fundamental to understanding the evolution of emotions (Bound-Alberti, 2019) and the dynamics of cultural forces that affect emotional experiences (Csordas, 2015). According to Kuppens and Verduyn (2017), emotional dynamics aim to understand the trajectories, patterns and regularities related to how emotions fluctuate over time, as well as their underlying processes and consequences.

## Research Question and Objectives

The main research question of this study is as follows: How have sociotechnical and relational transformations affected the experience of loneliness in young people residing in southern Spain during the Covid period? This can be subdivided into two research objectives:

1. Understanding how sociability and young people’s modes of interaction have affected the experience of loneliness in young university students in southeastern Spain during the Covid period.
2. Analysing how sociocultural dynamics affect and favour loneliness in Spanish youth.

Studies on loneliness in southeastern Spain—as well as in other areas of southern Europe, such as southern Italy—have been largely based on the fact that these are places that have maintained relatively more collectivist and communitarian forms of life, with close relations to northern Spain and Europe Hofstede Insights (2021). More individualistic societies have evidenced less loneliness because it is more normalized (Swader, 2019). In these areas, during the years that witnessed pandemic-related measures in which practically all social relations were virtual, people’s forms of social interaction were particularly affected and limited.

## Methods

### Participants

The participants were 40 young university students between 19 and 24 years of age earning a social education degree from the University of Almería, where one of the authors of this article works as a teacher. The group of participants was markedly female (6 men and 34 women), consistent with the profile of students earning a social education degree. Regarding the migration status, 4 young people were of foreign origin. Regarding hometowns, 11 students were from towns, 11 were from medium-sized municipalities, and 18 were from urban contexts. The occupations of family members indicate low socioeconomic conditions; fathers are mostly employed in the agri-food and construction sector, and mothers are unemployed or employed in the care sector. 29 young people acknowledged not living with their families during the research, being residing with roommates or alone. 31 recognized themselves as atheists (Table 1).

**Table 1:** Sociodemographic characteristics of the participants

Characteristics	*n*	Proportion (%)
Age group (years)		
19–21	26	65.0
22–24	14	35.0
Gender		
Male	6	15.0
Female	34	85.0
Birth place		
Foreing country	4 (1 Brasil, 2 Morocco, 1 Senegal)	10.0
Other provinces of Spain	19	47.5
Almeria	17	42.5
Type of place of residence		
Village (rural areas)	11	27.5
Medium-sized municipality	11	27.5
Urban city	18	45.0
Living with their family		
Yes	11	27.5
No (apartment sharing or living alone)	29	72.5
Religion		
Muslim	3	7.5
Catholic	2	5.0
Lapsed Catholic	4	10.0
Atheist	31	77.5

### Method and Procedure

For data collection, photo elicitation interviews (PEIs) were conducted. This method relies on the use of images or photographs to develop the interview. In this study, photographs representing or symbolising experiences of loneliness were selected by each student, and the interviews were based on the selected pictures (Clark-Ibañez, 2004; Copes et al., 2018; Epstein et al., 2006).

In February 2022, the project was presented to the potential participants, namely young university students. Those who volunteered to participate were asked to take approximately four to six photographs that they associated with their experiences of loneliness up to the day of the interview. The interview was structured into three sections: (1) introduction and sociodemographic data, (2) experiences of loneliness through photographs and (3) loneliness as a social problem in young people. In the first section, all participants were asked about their parents’ occupations, their religiosity and spirituality and the frequency with which they had experienced loneliness (offering the following as possible answers: “never,” “temporarily” and “permanently” for those who had felt loneliness in almost all stages of their life). In the second section, the conversation focused on describing and interpreting the images the participants had collected. In the third section, they were asked about loneliness as a possible target problem for young people. The interviews were conducted in an office at the university between March and June 2022 and had durations ranging between 50 and 90 minutes; all interviews were recorded and later transcribed. Approximately 2800 minutes of recording and over 500 pages of transcription were generated.

In Spain, the Covid pandemic is officially considered to have ended in July 2023. The state of alarm and home confinement ended in May 2021. However, social distancing and mask-usage measures were maintained until April 20, 2023. This was the participants’ first face-to-face course after two distance-learning courses.

### Data Analysis

After manual transcription, qualitative analysis was performed using Atlas Ti.22 software. The content analysis of the interviews and images was performed at 2 levels: descriptive and conceptual. The coding process followed the classic pattern of open, axial and selective coding rounds and subsequent inductive categorization was used as proposed by Glasser and Strauss (1967/2002), coinciding with the Noticing-Collecting-Thinking (NTC) system (Friese, 2019) (Table 2).

**Table 2:** Themes, categories and codes from content analysis

Themes by objective	Categories	Codes
Neoliberal sociability and loneliness Objective 1: to understand sociability and young people’s modes of interaction and their association with loneliness	Instrumental relationships	Having many relationships (hyper-linked mandate); interchangeability and replacement; simple and comfortable relationships; relational equivalence (receiving what is given); summative logic (add and contribute, never subtract); utilitarian relationships; detachment; obligatory reciprocity; self-concern; easy breakups; establishing trust without conflict
	Cold, scarce and dependent intimacies	Lack of empathy; ephemeral relationships; rejection of calls; toxicity and co-dependency; mistrust; cold and low-intimacy relationships; positive view of the non-complexity of virtual relationships; face-to-face relationships seen as exhausting
	Horror vacui: hyperconnectivity and hyperactivity	Constant need for contact; hyperactivity and hyper-verbalization; collapsed and saturated mind; hyper-connectivity; increased sociability but more loneliness
	Fantasy of omnipotence and independence	‘The most important thing is me’ (individualistic mandate); omnipotence; illusion of self-transformation; independence; ‘They are not like me’; identitarianism; selfishness; individualism; invulnerability; feeling of being able to influence others
	Culture of positivity, overcoming and self-esteem	Self-esteem as ‘loving myself’; ‘I am fine with me, and I am enough’; positivity; self-focus; self-improvement; ‘focusing on oneself’ dominates; culture of self-help
Loneliness cycle Objective 2: to understand the sociocultural dynamics that affect and favour loneliness	Phase 1: conditions favourable to loneliness	Feeling of constant judgment, hypercriticism and social pressure; competitiveness; individualism; isolation; being worth what one appears to be worth; demand for reciprocity
	Phase 2: hostile feelings associated with one’s environment	Not feeling enough qualified, living up to expectations or being worthy; fear and embarrassment in the face of judgment; boredom; need for validation; feeling different to stand out; suspicion of others; guilt
	Phase 3: defensive and protective reactions	Mistrust, deception and distancing; control of relationships by virtualizing them; avoiding dependence; leaving the comfort zone; pretending and holding back; never venting to others for fear of betrayal
	Phase 4: seeking shelter and protection	Protection from harm and judgment; virtual sociability; search for comfort; refuge in home and family; refuge in a group or among peers; refuge in co-dependent relationships; refuge in oneself and withdrawal inward; search for entertainment to stave off the emptiness; video games with virtual identities; refuge in psychologists
	Phase 5: social, relational and psychological effects of loneliness	Loss of social skills and the ability to interact; not knowing how to relate or communicate; virtual hyper-connection; cell phone addiction (being hooked); mental fatigue; mental disorders
	Phase 6: getting used to loneliness	Learning and normalizing loneliness; voluntarily isolating oneself; feeling proud of one’s autonomy and independence

Due to the dimensions of the research objectives, the analysis and interpretation of results has not focused especially on the role of sociodemographic factors (gender, social class, migrant status or rural/urban residence). This does not mean that they are not important, but that they should be analyzed in greater depth in future research.

### Ethical Criteria

This study was approved by the research ethics committee of the Universitat Rovira i Virgili. The committee, having evaluated the characteristics and ethical documentation related to the project, issued a favourable report. Likewise, the ethics committee of the University of Almeria was notified of the approval.

### Reflexivity and Posicionality

The interviews were conducted by the undersigned researcher who is a young, childless woman who teaches at the same university as the participants. Her role as a teacher at the same institution and her personal interest in the topic of loneliness may have had an impact on the dynamics of the interviews, influencing the way in which certain topics were addressed or omitted.

## Results

### Difficulty in Narrating Experiences of Loneliness

Of the 40 participants, only 2 denied having experienced loneliness. Of the 38 participants who had experienced it, 8 (20%) recognized it as sustained or permanent, while the other 30 recognized it as episodic, associated with experiences such as bullying, migration, moving from a rural to an urban locale, forced isolation, and the breaking or loss of ties. They also recognized an increase in social loneliness linked to the worsening of the quality of many of the ties they maintain, in which they “cannot be themselves” and which they distrust or find trivial or superficial. This worsening of relationships was associated with both unwanted social loneliness and desired loneliness by feeling liberated from relationships of dependence, pressure and obligation.

The youth participants agreed that loneliness is still taboo and is either not verbalized among people in the environment or verbalized only with people close to them. One of the reasons that makes it difficult to talk about this experience is the negative connotation socially attributed to it*.* One said, “It’s not easy to talk about. People do not admit that they feel alone for fear of being sidelined and considered strange. People tend to move away if they see your weaknesses” (P2). Recognizing oneself as a person who feels loneliness is associated with being odd, lacking social skills and experiencing relational deprivation and is interpreted as a form of social failure due to a deficit in social capital. One said, “Saying ‘I feel alone’ is a taboo. Young people are in a moment in which we are well, but we feel bad. We are not capable of saying those things in person because it means being different, not being like everyone else” (P4).

Likewise, the narratives reveal evident transformations in the modes of social interaction that young people themselves associate with their experiences of social loneliness, as described below.

### Neoliberal Sociability: Sociocultural Transformations in the Modes of Relationships

For the participants, the advancement of the internet and the use of new portable digital devices are the factors that have most transformed their ways of relating to each other. According to their stories, sociability is marked by the presence of two contradictory social mandates: on the one hand, the importance of generating and making visible links (**hyperlinked mandate**) “It’s like you have to show that you have many friends, that you are very popular and that you are followed by many people in social networks ” (P9) and, on the other hand, the mandate of independence, detachment and self-construction (**individualistic mandate**) “I notice that we care less and less about others and we are focused on ourselves. People only talk about themselves, about what is happening to them, and do not listen to others.” (P191). This contradiction generates constant conflict between accumulating contacts or breaking them to focus on oneself. The way to multiply links in the digital age is by generating individual virtual value, for example, as influencers. This idea is supported by the assumption that the greater the capacity to accumulate and make visible relationships is, the greater the social and personal value. Social value can accumulate and be obtained through erotic, aesthetic, creative and social capital, for which it is essential to focus on building an improved version of oneself. This approach assumes that the self and the body are mouldable**,** plastic and modifiable realities that are barely subject to biological, social, cultural or economic conditions, which can be improved with effort. This belief redirects young people from relationships with others to focus on themselves. One said, “I think the climate is cold. We worry less about others and are always focused on ourselves, only on being better. We are talking, and everything is: ‘me, me, me’” (P31). However, for many, the demands to increase their personal value are so intense that they become enormously frustrated and desire isolation, feeling unable to satisfy such expectations.

The participants recognized five characteristics of current social relationships that are especially important for explaining loneliness: the establishment of instrumental relationships; the development of cold, distant and dependent forms of intimacy; a need for hyperconnectivity and hyperlinking; a fantasy of self-construction and independence; and a culture of positivity that hinders the public or virtual expression of negative emotions.

The interviews describe in detail how usefulness regulates a large portion of their relationships, based on several principles, including on the one hand, *relational equivalence*, “to receive the same thing that is given”, and, on the other hand, an additive logic in social connections, by which the *relationships must* “add and contribute, never subtract”. Thus, a utilitarian calculation prevails in which when there is an imbalance between what is given and what is received, the participants opt to distance themselves or break up relationships, thus ridding themselves of what does not benefit them or what they consider *toxic*. One said, “Thanks to the pandemic, I have gotten rid of many toxic people who did not do me good, people who did not care and did not contribute anything” (P16). Sometimes motivated by enthusiasm for new virtual contacts (usually idealized), or by the fatigue of caring for ties that are not always satisfactory, or by the conviction that certain attitudes should not be tolerated, they recognize that they make and break many relationships quickly. Interchangeability and relational replacement are natural parts of relational dynamics.

The demand to establish a large number of links implies that most relationships will be shorter, less intimate and weaker than in the past. One said, “Forty years ago, you had a partner, and you would say ‘‘it will be for life’. In our generation, you know that relationships do not last forever, and in 99% of cases, it is like that” (P38). Likewise, it is assumed that both romantic partners and friendships are temporary and ephemeral, while only family ties are permanent. One said, “Friends may not be there from 1 day to the next. It scares me to share a lot of things because they will spread it around. If I tell my mother something, I know she will never tell anyone” (P33).

The dissatisfaction generated by cold and precarious bonds feeds what they describe as hyperactivity and the need for constant contact. Most university students admit that there are very few hours in a day that they spend without maintaining contact. They commonly use the expression “fill the schedule” to refer to the fear of having empty time, blank or silent moments in the day. The need for entertainment and contact ends up being exhausting, tiring and unsatisfactory, which makes loneliness generated from disconnection sometimes be recognized as a period of rest. Additionally, there is a heaviness generated by face-to-face and physical interaction due to the impossibility of cutting it short immediately; most even prefer to send messages rather than hold verbal telephone conversations for this reason.

The demand, pressure and harshness exposed on social networks—including ideals to be imitated and daily abuse—are balanced by a need for individual reinforcement that generates a culture of positivity, self-esteem and self-improvement. “What has helped me a lot in spending those moments of loneliness I think has been having a good attitude and a strong sense of self-esteem and learning not to depend on others, that you have to value yourself” (P31). The most difficult performance in this culture of positivity is expressing those traits that make social identity vulnerable.

### Cycle of Loneliness: The Process of Getting Used to Loneliness

**Fig. 1:**
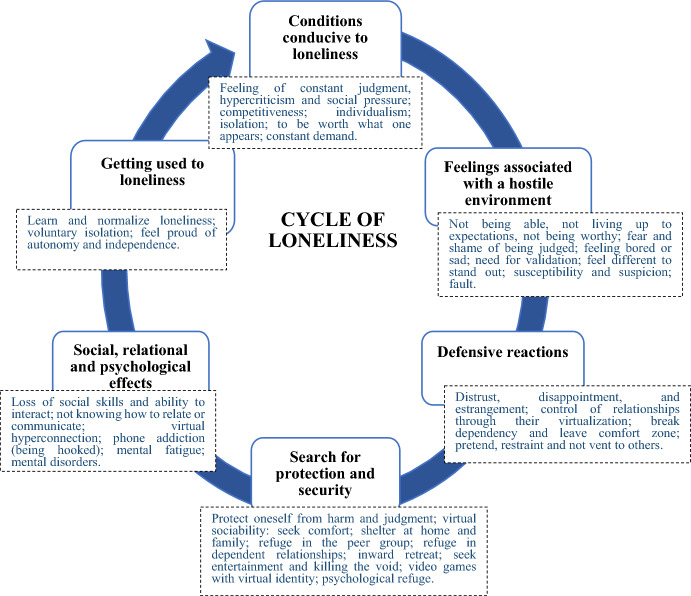
Cycle of loneliness: Sociocultural dynamics conducive to the normalization of loneliness. Own elaboration

Young people describe learning to coexist silently and individually with isolation and loneliness so that it is not emotionally negative or painful. Being independent and self-sufficient is perceived as an adaptive advantage to the extent that they feel less vulnerable in a hostile world. Under these conditions, there is a centripetal dynamic that feeds loneliness—both its experience and its normalization and acceptance. Figure 1 shows the cycle of loneliness (own elaboration). The process is composed of six phases that, although represented linearly, overlap in time. First, there must be a context of hostility conducive to loneliness (favored by the consequences of the neoliberal and virtual sociability) that generates negative emotions and triggers defensive and protective reactions and a search for safe spaces. Defensive reactions consequently have social, relational and psychological impacts, reducing social interaction and favouring the normalization and acceptance of loneliness, which contributes again to a climate that is pro-loneliness (Fig. 1).

#### Phase 1: Conditions Conducive to Loneliness

As described in the first section, there is an arid social and virtual climate that is difficult for young people to bear and that they describe as fundamentally conditioned by high levels of criticism, pressure and the display of opinions and judgements*.* One said, “People are very used to criticizing everything. They see something they don’t like and, sometimes through social networks, say the first thing that comes to mind. They say it without a filter, without thinking if they can hurt someone” (P6). Social networks allow the amplification of criticism and the feeling of constant attack and threat, facilitated by anonymity and virtual personal distance.

To function in the virtual environment, young people recognize the importance of developing perfected and armoured virtual identities. However, the construction of a perfected self is difficult to achieve and maintain without being undermined by constant comparison and self-criticism at the global level, where someone better is always found.

I like to draw, but I get very frustrated because things do not turn out the way I want, and I end up leaving them. [In social networks], you always see how well someone from China draws, one from Russia... the really good ones and, although many people don’t know how to draw, you end up only seeing the good, the handsome, the athletes, and so on. So, when you get something out, maybe it’s not even gone wrong, but it didn’t come out as you’d pictured it in your head and you feel really bad. This could also happen before, but I think that as time goes by, it is getting worse. As globalization is greater, you always make something and then compare yourself. Of course, that makes everyone frustrated. There is always someone who will be better in everything they do. (P34)

The constant perception of pressure and self-demand online and offline causes young people to describe standards that are very difficult to meet. They must constantly demonstrate meeting their goals and achievements, which translates into external and introjected pressure: “No, actually, the pressure is caused by myself. That is the problem” (P21). Although they sometimes rationalize the fictitious nature of networks, it is impossible to escape a dynamic as hyperactive and hyperdemanding as the virtual one. “I demand more of myself because I follow a lot of people who always think that I should be doing what others are doing in that video.” (P8)

The algorithms of the networks direct the gaze on certain aspects appropriate to the profiles of the consumers, making normality and mistakes invisible. Constant and global exposure generates a visual culture in which the canons of aesthetics, beauty, creativity, authenticity and sociability take on central importance. One said, “When you are physically attractive, it does not matter where you are, where you come from, that person will succeed. This is very clear on Instagram. When you are a guy who physically does not attract much attention, like me, everything will be much harder” (P22).

#### Phase 2: Hostile Feelings Associated with the Environment: Not Living Up to It and Seeking Validation

The experience of a hostile environment causes young people to develop feelings of self-doubt, of disempowerment, of not keeping up with expectations and of not being worthy. One said, “My mind is always wondering if I am doing it wrong. In the end, you even blame yourself for what you’re doing and how you’re feeling” (P6). The self-reflective loop on their own actions, constantly aware of the impact of their behaviour—as if within the panopticon—led them to regulate and inhibit their behaviour: “I feel like ‘did I say anything’, since I didn’t say anything, I don’t know, but let’s check if they’ve done something... and sometimes you feel guilty about things that don’t happen because maybe you didn’t do something” (P9). In fact, visual overexposure and constant comparison do not always translate into an increase in competitiveness; for some young people, this social competition is paralysing, and they describe impotence in the face of the impossibility of complying with these mainstream canons, experienced as a threat of isolation and disability. Thus, they withdraw to hide negative feelings or loneliness. One said, “Because sometimes I am afraid that the person will reply badly, take it the wrong way, stop being a friend or pass me by. Therefore, I usually keep things quiet” (P9). “If I feel like I’m going to be a bother or whatever, I’ll solve my problems on my own” (P3). In nonintimate friendships, they feel an obligation to reflect strength and well-being. When expectations are not met, many recognize feeling ashamed, responsible for their shortcomings and guilty of not being as expected. One said, “Of course, thinking ‘they have them, and I don’t’ will occur for some reason, they may be more sociable... I am different, and that is why I don’t have friends [...]. She blamed me that if I was alone, it was because of me” (P16). Under this pressure, they seek approval through virtual likes or positive feedback, which symbolizes admiration and recognition of their worth. One said, “It’s sad that a person is posting every 2 or 3 minutes what they’re doing to get a like because they constantly need approval, but it keeps happening to me” (P1).

#### Phase 3: Defensive and Protective Reactions

Hostility and fear of the judgement of others generate a series of defence mechanisms, including pretending, distrusting others, feeling disappointment, distancing oneself, seeking control of relationships and breaking dependence. In addition, the amount of information and proliferation of virtual communities also make it difficult to experience feelings of belonging. One said, “As there are more and more opinions, more varied, it costs more to find your place” (P38). This context makes some people recognize themselves as a suspicious generation, on the defensive, with a predisposition towards reactive criticism.

Therefore, many recognize maintaining online and offline behaviour based on pretend performances or selective displays of only certain aspects of reality. One said, “You have to spin a life that many times you don’t actually have” (P2). The fear of being judged makes it difficult to show oneself naturally to others.

Young people have more loneliness, but they increasingly hide it. This is because of social networks. That’s why saying: “I don’t want them to see my fears and my bad things; I want them to see my good things, to see me with people, to see me laughing and having a good time”. So they try so hard to do that that they also do not deepen any relationship, nor in themselves, and then, they are alone, although they try not to be, and when they are actually alone, they can’t endure it and then have to look for someone, even if it is to stay, even if they don’t know it, because they can’t stand it. (P34)

The fear of the opinion of other people influences each public act. One said, “When you are in a classroom or you are uploading content on networks, you are always thinking about people’s opinions” (P8). This climate of criticism, pressure and demand increases the distrust of others. It is assumed that peers and friends will speak badly of them and criticize them. One said, “I don’t trust people very much because they can always betray you or brush you aside at the slightest bit of change” (P10). Young people agree that trust makes them vulnerable and exposes them to harm that can penetrate them and that they must, therefore, protect themselves by maintaining distance. One said, “If I trust, they can hurt me, and I do not want to suffer” (P10). Faced with distrust due to fear of betrayal, judgement, dependence or harm, many young people choose to maintain control over their relationships and minimize the feeling of dependence, where the person is exposed to harm, pain and judgement.

#### Phase 4. Search for Shelter and Protection

The defensive reactions of distrust and fear generate distancing; additionally, other spaces of protection are sought in different environments. Some young people choose to take refuge in their family, others choose to do so in a smaller group of peers or in intimate relationships, and most also say they take refuge in themselves and in virtual environments. In all cases, retraction into smaller and closer concentric circles is observed. When they recognize a return to family contacts and the warm environment of the home, the most prominent figure is that of the mother (or simply the mother’s home as a place of protection), on whom the responsibility of care falls: “Many times when I get those lows, it’s true, I say to her ‘Mom, I feel alone, and I feel bad’. My mother always tries to cheer me up” (P11). In more rural environments, the role of grandparents also stands out.

Sometimes, the lack of ties and the feelings of helplessness manifest in the form of remaining in harmful intimate relationships. There is a fear of breaking all ties; in particular, women remain in romantic relationships under blackmail with the threat of loneliness or of being alone. One said, “I had a very toxic relationship, and that did not suit me, but I clung to that person because I felt alone and said: ‘If I lose them, I lose everything’ ” (P11).

For the majority, there is also a search for refuge around themselves. After processes of change or suffering, they recognize internal withdrawal, a need to be with themselves. Many learn to accompany themselves. One said, “I am not alone; I have myself. My family is a support, but the real support is myself. [...] At a stage when I was sick, I asked myself if God or my family would help me, but I thought: ‘You are here, you are enough for you’” (P10). When this happens, young people recognize that their interest in leaving the comforts of home disappears, postponing interactions with others and making them unwilling to meet people. One said, “Before, I wanted to party, but with the pandemic, I got used to not going out, and I want to be alone at home. Sometimes, if my friends call me to go out, I lie to them and say I have to study” (P7). However, they are aware of the negative effects of their isolation and withdrawal. One said, “You try not to think about it, but it affects you negatively because you become more and more isolated, you become more closed, antisocial and you get stuck in your world” (P31). In many cases, withdrawal is linked to an increase in the use of social networks and video games. Virtual socialization temporarily curtails feelings of criticism by allowing the individual to hide their physical aspects or their identity, by remaining anonymous or displaying another self, thereby avoiding the emotional flood of worry, pressure and disappointment.

#### Phase 5: Social, Relational and Psychological Effects

Emotional, defensive and protection-seeking reactions have social, relational and psychological impacts. The most repeated outcome of desocialization is a decline in social skills. This is explained by multiple reasons. One reason is forgetfulness and the lack of practice in mimicry and gestural expressions. There is even reference to a postpandemic lockdown. One said, “We have spent so much time without being able to put a face to a person and that person knowing your emotions, that now it is difficult for us to express emotions. Even people who used to be effusive now seem to be fading” (P4). For many, home confinement and distancing have meant a sudden change in their way of being and relating, becoming more serious, colder, more distrustful and reserved: “Now that things don’t matter so much to me, I’m more reserved” (P31). For those who already had relationship difficulties, the context has made them worse: “I am noticing that it is getting more difficult for me to relate. It’s even hard to hug others” (P28). There is also a lower tolerance for spending time with people. After a period of contact with people, they need more time to rest. One said, “When I am with many people, there comes a point that I’m done with socialization and I become boring to be around, and I leave because I no longer feel like talking; it is difficult for me to bring up conversation topics, and being with so many people overwhelms me” (P33). Some more extreme cases have drastically reduced the degree of interaction with people, which is a source of concern for people in the environment.

These social and relational aspects feed the compulsiveness already described in the search for relationships and contact because any relationship that is maintained does not continue to satisfy or satiate the need. Difficulties relating and communicating in person are associated with greater addiction to mobile phones but also with greater burnout and mental fatigue.

#### Phase 6: Getting Used to Loneliness

In the scenario described by participants, loneliness and voluntary isolation are manifestations of strength and independence. Eluding all sources of harm and conflict and avoiding confrontation are seen as the main strategies of power. Independence is lived with pride. Young people also describe a need to find valuable lessons around that experience: “I thought about how strong I was making it by myself alone, how proud I was of myself. I was even crying with happiness, saying yes, bad things can happen in life, but you will always end up learning something positive” (P7). There are numerous accounts that emphasize the need to accept and enjoy solitude, usually when it is transitory or episodic, because it is understood as a moment of rest, learning or personal growth. On the other hand, for those in whom solitude is permanent or chronic, loneliness is naturalized to the point of generating a certain indifference: “Since I have always been alone, it is not overwhelming; it is something that has always happened to me. I can point out more moments in which I have not felt loneliness than in those that I have.” (P8). There is also a third, very large group of young people who recognize that while loneliness is a necessary path to maturity, it is painful and is spoken of with suffering. One said, “For me, being alone is the worst. Since I came to the city, I have thought about it a lot, and I say, but why, if after all, loneliness is something you have to have in life? Little by little, I am learning, but I have ended up calling acquaintances to tell them: I feel very alone; it scares me” (P23). Likewise, another student observes:

I don’t know, there was a summer when I was not working and I was not studying and my parents had gone to Morocco and I was home by myself; I was alone, and I had no friends. So, I picked up my phone and saw that I had no one to write to on WhatsApp. That afternoon, I cried with helplessness. I know it seems strong, but I truly don’t have friends, and I’m not being dramatic; I’m being really natural, and I consider myself very mature (P22).

The increase in the experience of loneliness is linked to a growing social demand for isolation. One said, “I believe there is a shared need to isolate oneself. The old way we would say ‘I’m going out’ before is now ‘I’m going home now’” (P4).

## Discussion

Our interviewees recognized loneliness as a form of social suffering in young adults (Cigna, 2019; Fox, 2019; Kleinman et al., 1997). Ninety-five percent of the participants acknowledged having experienced both undesired and desired loneliness at some point in their lives, a percentage that is higher than that reported in the study by Qualter et al. (2015), and 20% expressed constant or chronic loneliness, consistent with the results reported by Dulmen and Goossens (2013). The data from this research—despite being qualitative and not generalizable—reinforce two ideas: on the one hand, there is a high awareness and recognition of loneliness both as a personal experience and a social problem, and on the other hand, a qualitative strategy can be effective in investigating a situation that is still recognized as taboo, as already pointed out by Fromm-Reichmann (1957). Thus, loneliness continues to appear as an indicator of fragility, a manifestation of social disability, which is hidden behind the projected shield of a strong and invulnerable self in an uncertain world (López-Mondejar, 2022).

In accordance with our first research objective, we determined that the forms of neoliberal and virtual sociability found in the young Spanish participants had a profound impact on the experience of loneliness, which was especially strong in contexts in which offline community ties existed (as is the case in southeastern Spain). Among the aspects that explain this relationship (i.e. sociability-loneliness) is the conflict between the hyperlinked mandate and the mandate of independence and self-construction. The hyperlinked mandate can be connected to Ubieto-Pardo and Pérez-Álvarez’s (2018) idea of a regime of the “hyper” that constantly requires children and adolescents to maximise their production and consumption in all spheres of life, in line with the excessive amount of stimuli, images and demands to which they are socially exposed. The pressure for social performance conflicts with the mandate that drives them to focus on the construction of themselves for their entire lives. There is not enough time for one to focus too much on oneself and have too many new relationships at the same time. As Illouz (2007) points out, the demand for hyperconnection and virtual hypercontact, with short and intense relationships, is recognised as one of the aspects that prevent the intimacy demanded by romantic relationships, friendships and family. For the participants, romantic relationships, friendships and family relationships (to a lesser extent) were sources of distrust. The feeling of virtual exposure feeds feelings of judgement, criticism, objectification, constant evaluation and exposure, in addition to increasing suspicion towards peers and partners. In this study, the participants expressed the decrease in or lack of intimacy in different ways, such as bodily distance, incomprehension of gestures, suspicion towards comments, distrust, a threat of betrayal, a lack of empathy and perceived rejection. Thus, many aspects related to bodily and linguistic expression are associated with an increase in loneliness, eliminating the sharp distinction between isolation and loneliness proposed by Peplau and Perlman (1982).

The sociocultural and technological characteristics to which young people are exposed seem to contribute to the normalisation of loneliness, which increases as loneliness becomes more accepted. This finding is consistent with studies indicating that contemporary feelings of loneliness are less frequent in more individualistic societies, not because they are experienced less but because they are more accepted and normalised (Heu et al., 2019; Lykes & Kemmelmeier, 2014). Other reasons that enhance the processes of internal retraction are: the disembodiment of interpersonal relationships and the creation of an idealised virtual self that is shielded from openness to the other (Guardiola, 2019; Zafra, 2010).

Regarding the second objective of this study, understanding the sociocultural dynamics that favour loneliness has made it possible to describe a sociocultural cycle of loneliness. This implies a vision that does not biologise loneliness as something inherent to adolescence/youth (Luhmann & Hawkley, 2015), reducing it to a neurobiological response. The importance given to social and technological pressure in the dynamics of young people’s loneliness has also been described in studies with young people from impoverished neighbourhoods in London (Fardghassemi & Joffe, 2021) and runs parallel with studies on hikikomori (Saito, 2013). Both are typical of neoliberal contexts in situations of socioeconomic crisis and major sociotechnical transformations. These social groups commonly exhibit an emotional repertoire linked to shame, distrust, betrayal and sadness under exposure and a feeling of tranquillity in refuge (Ozawa-de Silva & Parsons, 2020). The cycle bears some similarities to the neurodevelopmental proposal of loneliness developed by Hawkley and Cacioppo (2010), as both cycles explain the same tendency towards a progressive decrease in contact and an increase in hypervigilance in the face of stressful situations generated by loneliness. However, our cycle provides an affective bodily and sociocultural understanding of what Hawkley and Cacioppo explain as a neurohormonal response triggered by any instance of stress or threat.

### Limitations

A significant limitation of this study stems from the pronounced gender disparity among participants. Consequently, readers should interpret the results with an awareness of the substantial overrepresentation of women, highlighting the imperative for future research to address this imbalance by ensuring more equitable gender representation in analytical samples.

## Conclusion

In conclusion, the study illuminates the intricate sociocultural dynamics surrounding loneliness among southern Spanish youth, revealing a complex interplay between societal pressures, digital advancements, and individual experiences. Loneliness, though widely recognized, remains a taboo topic due to societal stigma, leading many to grapple with their experiences silently.

The rise of neoliberal sociability, characterized by a conflicting mandate for hyperconnectivity and individualistic self-construction, exacerbates feelings of disconnection and superficiality in relationships. Instrumental relationships further contribute to a culture of disposability, where connections are easily discarded if perceived as non-beneficial.

The cyclical dynamics of loneliness reflects a societal normalization of this experience, with young people adapting to its presence while simultaneously yearning for genuine connection. While some find solace and personal growth in solitude, chronic loneliness persists as a source of distress and suffering for many.
